# Knowledge transfer and innovation performance of small and medium enterprises (SMEs): An informal economy analysis

**DOI:** 10.1016/j.heliyon.2020.e04740

**Published:** 2020-08-16

**Authors:** Ayodotun Stephen Ibidunni, Aanuoluwa Ilerioluwa Kolawole, Maxwell Ayodele Olokundun, Mercy E. Ogbari

**Affiliations:** a Department of Business Management, Covenant University, Ota, Ogun State, Nigeria; b Center for Economic Policy and Developmental Research, Covenant University, Nigeria; c Center for Policy Research and Industry Linkages, Shaveh Consulting, Nigeria

**Keywords:** Knowledge transfer, Innovation performance, SMEs, Informal economy, R&D, Social networking, Entrepreneurship, Business policy, Management, Business management, Strategic management

## Abstract

SME operators in the informal sector of developing economies have a significant influence on their nation's economies through their involvement in international business relationships. However, the existing deficiency in the literature to show empirical relationships between knowledge transfer, from these SMEs and their international business partners, and innovation performance is a significant gap in the strategic management and international business literature. Therefore, this paper explores the link between knowledge transfer and innovation performance of informal economy SMEs that are involved in international business relationships. The study included a survey of 370 owners-managers and managers of small and medium enterprises in Nigeria's informal electronic market. Using Structural Equation Model (AMOS 22) this study shows that knowledge transfer dimensions, such as R&D and social networking, have varying levels of impact on innovation performance of informal sector SMEs. Knowledge transfer from training showed an inverse and insignificant relationship with innovation performance. The study established implications and recommendations that will be useful for theory and practice.

## 1 Introduction

The significance of knowledge transfer has become more renowned as a critical element in the micro, small and medium enterprises (MSMEs) sector. Knowledge transfer is the movement of knowledge through a/some channel(s) from one individual or firm to another (Abou Hashish, 2017). It also refers to practices that include the exchange of individuals' experiences and work-related knowledge (Tassabehji et al., 2019). Within international business relationships, such as with Home-based MSMEs operations and their foreign business associates, knowledge transfer involves both tacit and explicit knowledge exchange (Cerchione et al., 2015; Ibidunni, 2020). Explicit knowledge is any form of experience that is codified and can be stored through documentations for future reference while tacit knowledge is the use of intuition to discern issues (Hajidimitrious et al., 2012; Ibidunni et al., 2018a). Regardless of what type of knowledge is involved with an international business relationship, knowledge transfer is vital to the smooth operations of SMEs. This assertion is evident through its ability to help organisations to have an advantage on a wide range of skills and expertise, thus complementing new product innovative performance of firms involved in such knowledge-based relationships.

According to Zubielqui et al. (2015), SMEs can achieve innovation performance through leverage on knowledge transfer relationships with their foreign business partners. Although, this claim has a global concern for SMEs, within the context of the present study is particularly crucial to SMEs operating in Nigeria's informal economy, where the business value chain involves procuring products and services from business associates in foreign nations, like China and other Asian, European and American economies. Consequently, new product innovation performance will reflect in the way SMEs in Nigeria can make good use of the knowledge from their international business partners to become creative with ideas and transform into a producing nation with a higher quality of products, services and business processes. Innovation provides opportunities for growth in new markets, new ideas and new invention (Bessant and Tidd, 2007; Adegbuyi et al., 2018).

According to Baumann and Kritikos (2016), within the German region more than 90% of large companies report the introduction of new products innovation during three years, the share of medium-sized firms report innovation is about 80% and more than 65% in small firms. Small and Medium Enterprises in Nigeria are involved mainly with international activities where they transact businesses through (products and service) importation from foreign business partners based in other countries spread across European, North American and Asian countries. Nonetheless, very little exist in their business activities, especially in the area of innovation performance, especially new product innovation, and hence repositioning Nigeria among the league of producing economies, away from its present consumption stance. Also, it might suggest that there are minimal efforts by Nigerian SMEs to deliberately make efforts to engage in knowledge transfer activities, such as training, research and development, social networking and collaborations with their foreign business partners.

This research, therefore, raises a question about the capacity of SMEs operating in the informal sector of developing economies (for example, Nigeria) involved in international business relationships, to relate with knowledge transfer from their foreign business associates and to translate same into viable new product innovations. More obvious is the fact that there is a shortage in the existing literature that shows empirical relationships and that explain the processes between knowledge transfer and innovation performance in informal economies, such as Nigeria. Nevertheless, the importance of understanding the relationship between knowledge transfer and innovation performance within these developing and informal economies, like Nigeria, cannot be overemphasised. Nigeria, as a world's fast-emerging economy and the largest economy in Africa (Giles, 2018; PwC, 2019). Nigeria is a choice location for many Asian and European businesses and a fast-growing country for technology utilisation (Adi, 2015; PwC, 2016; Ogundana et al., 2016; Nigerian Communications Commission, 2018). Therefore, this study focused on investigating the link between knowledge transfer and innovation performance of Small and Medium Enterprises in an Informal Economy, such as Nigeria.

## 2 Literature review

### 2.1 Concept of knowledge transfer

Several studies in the field of knowledge management and strategic management have investigated different dimensions of organisation and management of knowledge in various economic and industry contexts (Liyanage et al., 2009; Trautman and Mckee, 2014; Mislan et al., 2016; Hassan et al., 2017). There are distinctive parts of knowledge management, which include knowledge creation, knowledge capture, knowledge sharing, knowledge application and knowledge transfer (Liyanage et al., 2009; Ibidunni et al., 2017). Findlay first introduced knowledge transfer in 1978, with descriptions as knowledge sharing, knowledge obtaining, and knowledge stream (Sheng et al., 2013). Different Literature views the exchange of knowledge as the transference of information from the root to the beneficiary in a given setting. Davenport and Prusak (1997) established that learning exchange includes two activities: transference (sending or introducing information to a potential recipient) and ingestion by a gathering of individuals or by an individual. If knowledge has not been transmitted or consumed for a desirable purpose, active knowledge exchange has not occurred.

Knowledge transfer or exchange involves organisational learning transits between a source to a beneficiary for achieving predetermined outcomes (Nguyen and Burgess, 2014; Hassan et al., 2017). In a broader sense, it incorporates employee willingness to interact with others (donate knowledge) and to also learn from them (collect knowledge) (Saide et al., 2016; Ibidunni et al., 2018b). Knowledge transfer has six stages: examining, codification, deliberation, dispersion, ingestion, and affecting. A knowledge transfer framework comprises of four classifications, in particular, the sender, collection of learning, approach, and accomplices or get (Wang et al., 2019).

### 2.2 Concept of innovation in an organisation

Innovation is the practice of conceptualising and processing of novel ideas that generate commercial values in the form of new products and services. Within the purview of business practices, innovations have an end goal of servicing customers' expectations in new ways (Jackson et al., 2013). It is a strategic organisational tool through which organisations manage change and establish a new market breakthrough (Ndesaulwa and Kikula, 2016).

Organisations innovate across several levels, including products, services, manufacturing designs, service delivery procedures, and managerial processes or in designing the organisational hierarchy (Omodafe and Nwaizugbo, 2017). However, and most often, innovating is associated with the production, to meet new customer requirements; and with processes with a focus on improving on procedural efficiencies and effectiveness. According to Scholars like Mwangi and Namusonge (2014) and Shinnar et al. (2018) innovation is critical to the growth and competitiveness objectives of any organisation, be it small, medium or large. Besides, most organisations consider innovation as an essential element to increase their profit and market share (Meroño-Cerdán and López-Nicolás, 2017; Shujahat et al., 2019).

Earlier research conducted revealed that innovation positively affects the performance of firms (for example; Hewitt-Dundas and Roper, 2018). A study carried out by Ndesaulwa and Kikula (2016) revealed that innovation should be widely encouraged across the country and organisational levels because of its role in promoting economic worthiness. This argument rests upon the fact that innovation proves the firm or nation's dynamic capability, especially concerning managing environmental shocks (Oludayo and Ibidunni, 2019).

There is much argument about the types of innovation. Still, the primary focus surrounds Schumpeter's (1934), four types of innovation they are product innovation, process innovation, market innovation and organisation innovation (Tavassoli and Karlsson, 2015). Products and process innovation are closely related to technological innovation, while market and organisation innovation is in the category of non-technological novelty (Abel-Koch et al., 2015).

## 3 Hypotheses development

### 3.1 Training and innovation performance

Ibidunni (2019), suggested that to an extent, organisational success rely on its human resource capabilities. Employees with high levels of exposure to the job or business-related training are considered a cornerstone for such success. More so, in the present global economy organisations are faced with increasing levels of competition, rapidly evolving technologies and highly dynamic operating environment. Also, the effects of Globalization and the evolving customer preferences have their tolls on stiffening the operational procedures in the business economy. Consequently, organisations, and especially SMEs, must keep abreast with changing patterns through training. According to Ibidunni et al. (2017) intense competition and the rapidly changing market environments, cause firms to find ways to capture or create knowledge that can improve their products or develop new products to satisfy customers in other to gain a competitive advantage in the industry.

Consequently, employee training is perceived to be a viable channel to new knowledge creation and transfer that will help them develop the required innovations (Blome et al., 2014; Zou et al., 2018). Regular training guarantees entry to outside knowledge, and it expands a firm's tendency to innovate (Falck et al., 2009; Ibidunni, 2019). Consequently, this study hypothesises that,

H1 Knowledge transfer acquired through training enhances the capacity of informal economy SMEs' for improved innovation performance.

### 3.2 R&D and innovation performance

Studies have shown that knowledge sharing based on R&D is a critical activity for the success of SMEs' innovation performance (Gupta and Wilemon, 1991; Fain et al., 2011; Shim et al., 2016). This is without prejudice to how R&D knowledge flow is channelled in the organisation, whether it be from diverse or controlled sources (Cuervo-Cazurra et al., 2018). According to Frankort (2016) firms make the most of R&D knowledge transfer for innovation when the firms have established an active partnership in similar technology domain, while yet operating in different product markets. Generally, small and medium enterprises that are highly profitable with innovations appear to incur some percentage of more cost on R&D than those that do not (Baumann and Kritikos, 2016). Thus, validating the payoff in R&D expenditure as a way of driving SMEs' innovation performance. The assertions of Adomako et al. (2019) support the fact that R&D intensity on successful innovations is better achieved through international R&D relationships.

Consequently, explaining the importance of R&D as a fundamental mechanism of knowledge transfer among partners in a global business relationship. Within the Nigerian SMEs industry, a study of this nature is critical to provide empirical evidence for the relationship between R&D and innovation performance, because very minute R&D activity exists within the SME sector (Olughor, 2015; Adelowo et al., 2017; Booltink and Saka-Helmhout, 2018). The limitations associated with limited R&D derives from their lack of human and financial capital to execute the enormous tasks involved with R&D (Baumann and Kritiko, 2016). However, this study anticipates that empirical evidence of this nature would instigate a positive response from the SME industry actor. Consequently, we hypothesise that:

H2 Knowledge transfer through R&D has a significant impact on informal economy SMEs' drive for improved innovation performance.

### 3.3 Social networks and innovation performance

**Figure 1:**
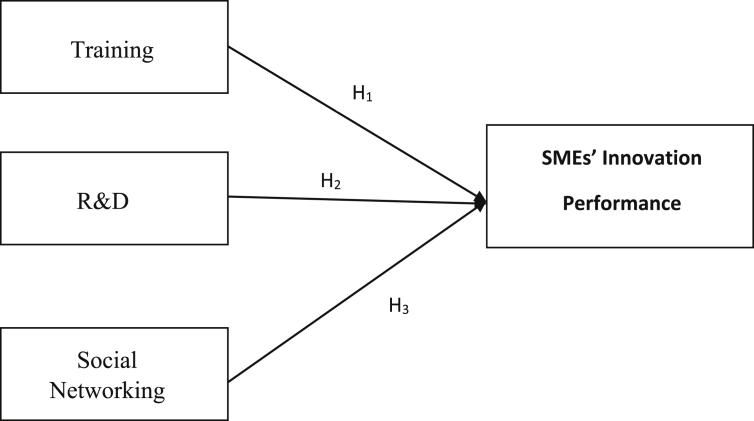
Conceptual model.

Social networking, drawn from the social integration theory, is a practice whereby social actors connect within a system governed by mutual interests and relationships (Beresnevièiûtë, 2003). Social networks are an aggregate of informal and formal relationships which entrepreneurs build with the environment that are internal and external to their context of operation (Hakimpoor et al., 2011; Jevwegaga et al., 2018). Social networking is a viable knowledge transfer indicator and a viable platform for aiding learning (Birley, 1985; Miller, 2005; Tian et al., In press). According to Hung (2017), the density of social networking among teams, such as a group of entrepreneurs, enhances their centrality of technology, which in turn could result in higher innovation performance for the firm. Ogunnaike and Kehinde (2013) argue that social networks are critical to entrepreneurial success because they give entrepreneurs access to knowledge and technology required for market and technology innovation performance (see Figure 1). Therefore, this study suggests that,

H3 Knowledge transfer acquired through social networking enhances the capacity of informal economy SMEs' for improved innovation performance.

## 4 Methodology

The research design adopted in this study is survey research. In the survey method, this study gathered opinions of respondents by the use of questionnaires. The population of this study was 100,000 owners-managers and managers of small and medium enterprises in Nigeria's informal electronic market (Ayeni et al., 2018). The informal sector of Nigeria, and especially its electronic market, is an economic sector that consists of businesses that are not fully regulated by governments and other institutions; they are businesses that are only partially registered and do not maintain complete books of account (Bank of Industry, 2018). This present research considers the informal sector as a very strategic aspect of the Nigerian economy that is worthy of research focus because the informal sector consists of more than sixty-five per cent of Nigeria's business economy and employs seventy-two per cent of Nigeria's economic population (Obi et al., 2018). In using the survey method to gather responses for this study, the descriptive approach of research established the link between knowledge transfer and innovation performance of Small and Medium Enterprises in Nigeria's Informal Economy. The descriptive design is of particular importance to this research agenda because it supported the study's aim of investigating the degree of the impact knowledge transfer dimensions on innovation performance of SMEs in the developing informal economy that is involved in international business relationships. To test the hypotheses and analyse the data for this study, SPSS and AMOS 22 was used to examine the goodness of fit criteria and the relationship between knowledge transfer and innovation performance of SMEs. The ideal conditions that apply to use Structural Equation Modelling AMOS 22 guided the execution of this study. For example, Loehlin and Beaujean (2017) suggested that a minimum sample size of 100, or where possible 200 and above, is suitable for establishing theories using AMOS. The 370 respondents that formed the basis for this study justify the appropriateness of SEM analysis for the study. Also, the preconditions surrounding the goodness-of-fit measures, for example, root mean square error of approximation (RMSEA), comparative fit index (CFI) and Tucker-Lewis index (TLI), as argued by Hair et al. (1998) and Byrne (2001) guided the analysis stage of this present study.

### 4.1 Measures

Within the MSMEs cluster, literature has shown the effectiveness of knowledge transfer along the supply chain to be the primary source of innovation (Corral de Zubielqui et al., 2019). Valdez-Juárez et al. (2016) examined the role of knowledge management and transfer dimension on performance of SMEs, with innovation as a mediating variable. Hence the central emphasis was on the economic performance of the firm. The fascinating insights from these existing studies have given credence to and elaborated understanding of the relationship between knowledge transfer and innovation performance (Susantya et al., 2012; Putra and Santoso, 2020; Erdin and Ozkaya, 2020). However, the present study demonstrates novelty by investigating knowledge transfer of SMEs that are operating in the context of a developing economy and are involved in international business relationships with foreign business associates.

Consequently, items in the research instrument adapted from existing research that closely related to the present study; and the research instrument items were modified to fit into the present study. Details on Knowledge transfer variables such as questions on training, including the exposure of SME operators to continuous development to acquire new skills on the job, were gotten from O'Regan et al. (2010). Questions on R&D, defined as the extent to which SMEs invest in R&D spending, were obtained from Hottenrott and Lopes-Bento (2014). Social networking items, defined as the identification of SME operators within a social network that enhances Intra and inter-industry knowledge sharing, were extracted from Beresnevièiûtë (2003); Tian et al., (*In press*). The dependent variable is new product innovation performance and is the ability of SMEs to mobilise technology and skill to innovate new products that meet customer demands. It adapted from Kesinro et al. (2018), Ibidunni et al. (2014).

### 4.2 Sampling

The sample size for this study derived from using the Bartlett et al. (2001) formula. Based on their sample size determination parameter, a sample size of 370 respondents was used for this study. The respondents consisted of owners-managers and managers of small and medium enterprises in Nigeria's informal electronic market. Table 3 depicts the demographic characteristics of the respondents for this study. For this research, the simple random sampling method facilitated the process that ensured that selection of respondents was made without human bias, hence giving every member of the population the equal chance of participating in the survey. However, during the time of administering the research instrument, respondents who declined to participate in the study were respectably excused and replaced based on convenience of other respondents.

### 4.3 Measurement models

This study tested for reliability using Cronbach's alpha test. It associates each measurement item to every other measurement item and finds the average. Cronbach's alpha tests the reliability of a multi-item scale. When the coefficient is either 0.70 or higher, it is acceptable in a social science research study. The reliability coefficient of items used for the present study is 0.871. Hence, the present study established that the scale items were consistent with the intended purpose of what the research set out to measure. Moreover, for the individual instruments the results showed the following: training (α = 0.852), R&D (α = 0.876), social network (α = 0.576) and innovation performance (α = 0.914). The values justify the reliability of the scale items because most values are greater than the benchmark of 0.7. Confirmatory Factor Analysis (CFA) confirmed the validity of the research items. According to Fornell and Larker (1981) and Hair et al. (1998), CFA must meet three important conditions namely, (1) all item loading should be 0.65 and greater (2) composite reliability (CR) value should be 0.8 and greater, and (3) the values of average variance explained (AVE) for each item should be 0.5 and above. Table 1 depicts the CFA results for items used in this study. The results indicate that all three conditions outlined above for an acceptable CFA is accomplished. All item loading is between 0.70 – 0.98; the CR values are within the ranges of 0.90–0.93, and the AVE values all fall within 0.83–0.86. See Table 1 for details.

**Table 1:** Confirmatory factor analysis of research items.

Measurement Items	Loadings	Indicator Reliability	Error Variance	Composite Reliability	AVE	Cronbach Alpha
**Training**						
T1	0.9360	0.8761	0.1239	0.9007	0.8344	0.852
T2	0.7880	0.6209	0.3791			
T3	0.7080	0.5013	0.4987			
T4	0.8870	0.7868	0.2132			
**R&D**						
R&D1	0.8490	0.7208	0.2792	0.90529	0.8397	0.876
R&D2	0.8330	0.6939	0.3061			
R&D3	0.8140	0.6626	0.3374			
R&D4	0.8620	0.7430	0.2570			
**Social Network**						
SN1	0.756	0.5715	0.4285	0.92197	0.8654	0.576
SN2	0.82	0.6724	0.3276			
SN3	0.884	0.7815	0.2185			
SN4	0.985	0.9702	0.0298			
**Innovation Performance**						
IP1	0.8130	0.660969	0.339031	0.93677	0.8617	0.914
IP2	0.8780	0.770884	0.229116			
IP3	0.8000	0.64	0.36			
IP4	0.8920	0.795664	0.204336			

This present study also ensured discriminant validity for the constructs. Discriminant validity reflects the extent to which constructs differ from one another. By the general rule, Fornell and Larker (1981) suggested that the AVE should be higher than the shared correlations between the constructs. As reflected in Table 2, the condition for discriminant validity is fulfilled in this study because all items are above 0.5 and are higher than the squared correlations shared between the constructs. This assertion was established by average variance explained values, as presented diagonally in Table 2. More so, the mean values ranging from 3.8 to 4.2, and the standing deviation values generally less than 1 indicates that respondents regularly share a common opinion that suggests high levels of agreement on the items covered in the research instrument. The statistics, also, reflects that there is generally a correlation between the variables of interest at p < 0.01 level of statistical significance.

**Table 2:** Discriminant validity of the research model.

	Mean	SD	*1*	*2*	*3*	*4*
Training	4.2696	0.82009	*0.783*			
R&D	3.9709	0.88173	0.348∗∗	*0.811*		
SocialNetwork	4.0063	0.86709	0.225∗∗	0.411∗∗	*0.597*	
InnovatPerf	3.8155	1.09751	0.013	0.211∗∗	0.246∗∗	*0.762*

*Notes:* 1. Average Variance Extracted (AVE) for each construct is presented diagonally.

2. The numbers 1,2,3,4 Training, R&D, Social Network and Innovation Performance.

∗∗ Correlation is significant at the 0.01 level (2-tailed).

## 5 Analysis

A total of 370 copies of questionnaires administered to respondents were filled correctly and returned by the respondents.

Table 3 shows the respondents' demographic characteristics. Out of 370 respondents, 298 are male operators, while 72 are female operators. Also, 54 (14.6%) respondents are below 20years, followed by 156 (42.2%) respondents between 21 – 30 years, 114 (30.8%) respondents between 31 – 40 years and 46 (12.4%) respondents are 40 years and above. Moreover, 190 (51.4%) of the respondents are single, 162 (43.8%) of the respondents are married, 18 (4.9%) are either divorced or widowed. 120 (32.4%) of the respondents have below 5 years' experience in the business, 150 (40.5%) of the respondents have 5–10 years’ experience in the business, 100 (27.0%) of the respondents have 10 years and above experience in the business.

**Table 3:** Respondents’ demographic characteristics.

Demographic Characteristics	Frequency	%
**Gender**		
Male	298	80.5
Female	72	19.5
Total	370	100.0
**Age**		
20 and below	54	14.6
21–30	156	42.2
31–40	114	30.8
40-above	46	12.4
Total	370	100.0
**Marital Status**		
single	190	51.4
married	162	43.8
others	18	4.9
Total	370	100.0
**Years of Operation**		
Below 5 years	120	32.4
5–10	150	40.5
10 years and above	100	27.0
Total	370	100.0

### 5.1 Test of hypotheses

This study relied on the goodness of fit criteria for any SEM-AMOS analysis as postulated by previous studies. As depicted in Table 4, RMSEA (0.043), TLI (0.974) and CFI (0.981) satisfied the suggestions as presented by Hair et al. (1998) and Byrne (2001). Also, the normed chi-square results (χ2/df) for this study was 1.681, which satisfied the cut-off requirement, as argued by Kline (1998). Therefore, the following values in Table 4 confirm this study's goodness of fit statistics.

**Table 4:** The goodness of Fit Statistics for the Study.

Index	Goodness of Fit Statistics	Recommended Values	Remark
Ratio of χ^2^/df	1.681	Less than 3 (p < 0.05)	Very Satisfactory
RMSEA	0.043	0.05	Very Satisfactory
TLI	0.974	Approaches 1	Very Satisfactory
CFI	0.981	0.95	Very Satisfactory
NFI	0.955	Approaches 1	Very Satisfactory
GFI	0.954	Approaches 1	Very Satisfactory
AGFI	0.929	Approaches 1	Very Satisfactory

Source: Compiled by Authors.

**Figure 2:**
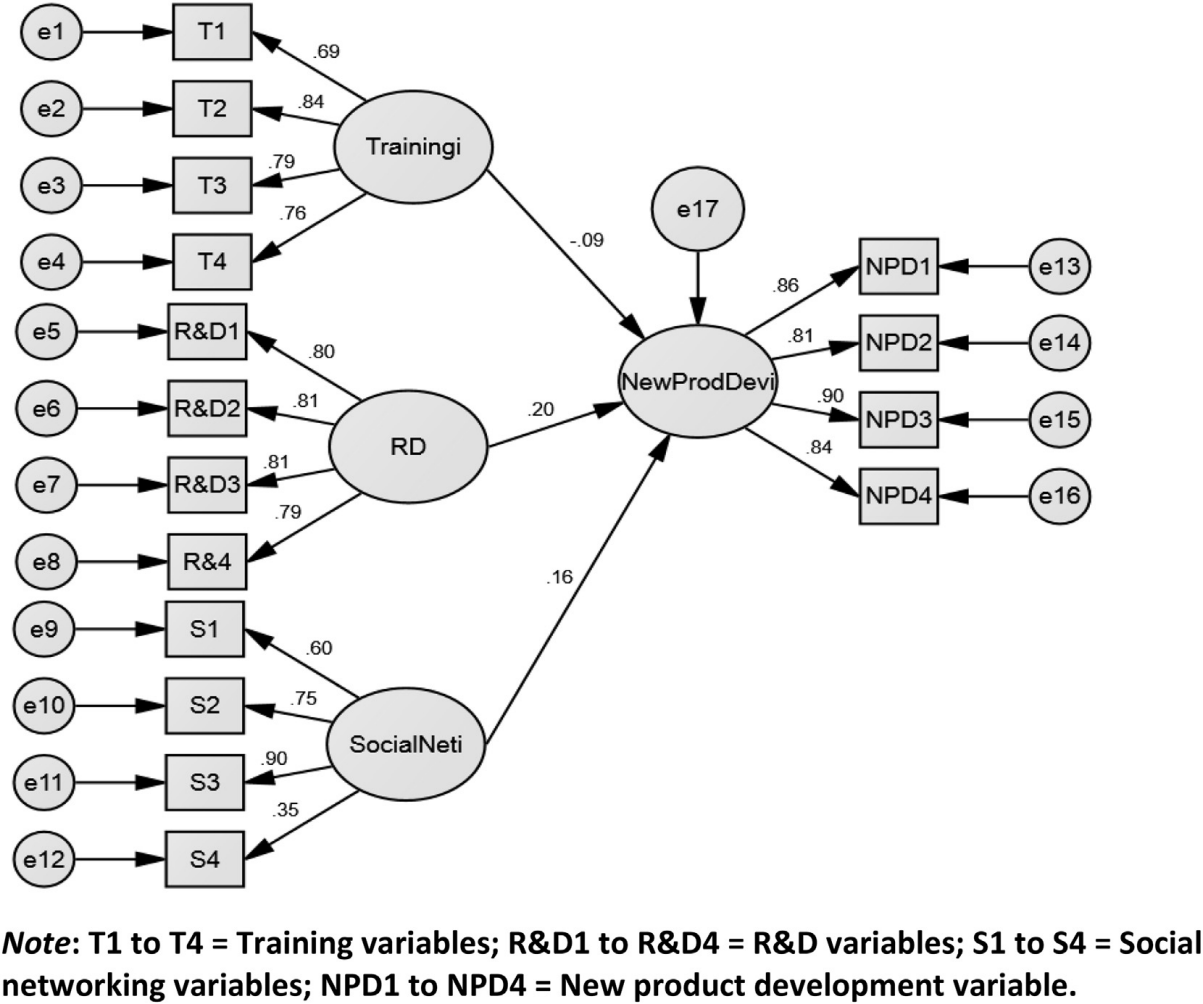
Path Diagram Showing the Hypothesised Relationships. *Note*: T1 to T4 = Training variables; R&D1 to R&D4 = R&D variables; S1 to S4 = Social networking variables; NPD1 to NPD4 = New product development variable. Source: The Authors (2020).

Figure 2 shows the path analysis for this study. The standardised form of regression results obtained from the statistically significant path analysis shows an effective relationship between knowledge transfer and innovation performance. The theoretical background with interpreting the standardised results from a regression relationship implies the change in the dependent variable as a result of a 1 unit standard deviation in the predictor variable (Freeze and Raschke, 2007). From the path diagram in Figure 2, R&D (r = 0.20) and social network (r = 0.16) demonstrated the strongest relationships with innovation performance. The importance of R&D and social networks to the new product innovation capability of SMEs involved in international business relationships is, therefore highlighted by these results. By implication, shifting activities and investments relating to R&D in the SMEs industry will result in up to β = 0.20 upward shift in the innovation performance of the SMEs.

Similarly, investments in social networks will move new product innovation performance of SMEs positively upward by β = 0.16. The relationship between training and innovation performance was negative and minute (β = -0.09). Although this relationship is contrary to expectation, yet it may be indicative of the fact that informal sector SMEs rarely engage in any form of training relationships with their foreign partners. Moreover, the result signifies that training does not make any significant contribution to the SMEs new product innovation performance. The results, also established that informal relationships and knowledge sharing acquired from social interactions, collaborations and strategic alliances built from social networks instead prove to be more effective than any procedural training activities.

The present result from this study is significant for improving the performance of Nigeria's SMEs sector. Currently, the SMEs sector accounts for about forty-eight per cent of national GDP and about ninety-six per cent of the total number of businesses in Nigeria, employing eighty-four per cent of the total population (PwC, 2020). Despite this, the SMEs sector is still primarily hindered in terms of maximising its potentials for growth and development (Wale-Oshinowo et al., 2018). The role of knowledge transfer for operators in the industry has received very minimal attention in existing research. However, in existing studies, the importance of knowledge sharing has been established to be critical to SMEs performance growth (Mislan et al., 2016; Hassan et al., 2017). The outcome from this study emphasises that the possibility of economic contribution, especially in terms of innovation performance, of SMEs involved in international businesses, can be improved significantly by focusing on social networks and R&D.

Table 5 shows the p-value indicators for all the relationships reflected in the path analysis. The Table reveals that all p-values for the reflective models are significant (sig. = 0.00, p < 0.01). The reflective nature of the structural model indicates that the changes in the latent variable results directly from changes in the indicators of such variable (Freeze and Raschke, 2007). The results in Table 5 shows that the reflective relationships of each construct were significant for Training, Social networks, R&D and new product innovation performance. Moreover, the results partially established the relationships between the knowledge transfer of SMEs and their innovation performance. The statistical result affirmed that R&D (sig. = 0.00, p < 0.01) and social networking (sig. = 0.00, p < 0.01) had significant impact on innovation performance. However, training (sig. = 0.133, p > 0.05) does not have any statistically significant influence on innovation performance.

**Table 5:** Regression weights for the relationship between knowledge transfer dimensions and SMEs’ innovation performance.

Hypothesised Relationships			Estimate	S.E.	C.R.	P	Remark
NewProdDevi	<---	Trainingi	-.122	.081	-1.503	.133	Not Significant
NewProdDevi	<---	RD	.245	.071	3.462	∗∗∗	Significant
NewProdDevi	<---	SocialNeti	.176	.067	2.633	.008	Significant
T4	<---	Trainingi	1.000			∗∗∗	Significant
T3	<---	Trainingi	1.098	.075	14.563	∗∗∗	Significant
T2	<---	Trainingi	1.070	.070	15.276	∗∗∗	Significant
T1	<---	Trainingi	.943	.074	12.800	∗∗∗	Significant
R&D4	<---	RD	1.000			∗∗∗	Significant
R&D3	<---	RD	.957	.060	15.847	∗∗∗	Significant
R&D2	<---	RD	.963	.061	15.860	∗∗∗	Significant
R&D1	<---	RD	.960	.061	15.624	∗∗∗	Significant
S4	<---	SocialNeti	1.000			∗∗∗	Significant
S3	<---	SocialNeti	.917	.146	6.266	∗∗∗	Significant
S2	<---	SocialNeti	.768	.122	6.276	∗∗∗	Significant
S1	<---	SocialNeti	.682	.115	5.923	∗∗∗	Significant
NPD1	<---	NewProdDevi	1.000			∗∗∗	Significant
NPD2	<---	NewProdDevi	.932	.048	19.233	∗∗∗	Significant
NPD3	<---	NewProdDevi	1.051	.047	22.505	∗∗∗	Significant
NPD4	<---	NewProdDevi	.983	.049	20.152	∗∗∗	Significant

∗∗∗ p > 0.05.

## 6 Discussion

This study focused on investigating the relationships between knowledge transfer and innovation performance of Small and Medium Enterprises (SMEs) in the Informal Sector of a developing economy, precisely Nigeria. Specifically, this research work raised three hypotheses from the review of the literature. The first hypothesis suggested a possible impact of training on innovation performance of the SMEs. Based on the results from the structural equation modelling analysis, training did not have a significant influence on SMEs' innovation performance. Hence, the emphasis on training as a strategic knowledge transfer tool for SMEs involved in international businesses may not necessarily enhance innovation performance. This result may suggest that SMEs operators in the informal economy of developing nations like Nigeria are more particular about real-life engagement with their businesses and learning through practical meetings with their international business associates that engaging with organised training programmes.

Moreover, the fact that most operators in this sector do not possess formal education could also be a resultant factor. The assertion is such that the SMEs operators in this sector have developed business skills through informal apprenticeships, rather than through organised training. Consequently, the result of this study demonstrates a different perspective from studies in more developed economies suggest that training enhances the survival rate of SMEs (Ibrahim and Ellis, 2003). The assertions of Terziovski (2009) also suggest that SMEs can achieve better organisational procedures and achieve higher levels of productivity by emphasising employee training. According to O'Regan et al. (2010), strategically focusing on training by SMEs grants them the leverage of accessing and utilising external and internal knowledge for the good of the organisation.

The second hypothesis for this study proposed an impact of research and development on innovation performance of SMEs operating in the informal sector. Based on the statistical analysis carried out, the result revealed that R&D positively impacted on new products development. Hence, SMEs in the informal economy of developing nations like Nigeria, which are involved in international businesses can support their innovation performance objectives by adoption R&D as a viable tool. Cummings and Teng (2003) asserted that R&D as a veritable tool of knowledge transfer works well for firms in international business relationships when each party well understands the essential aspects of knowledge to be transferred. R&D, in particular, generates new knowledge that is frequently the source of the firm's innovation performance (Grandinetti, 2016; Falola et al., 2020). Enterprises conducting R&D bring forth innovations and are generally the first to introduce new products, services and production processes into the market (Hottenrott and Lopes-Bento, 2014; Ibidunni et al., 2019). Evidence abounds that SMEs that conduct R&D make more profit from innovations than those that do not conduct R&D, and they also introduce new products to the market.

The third hypothesis was to identify the impact of social networking on innovation performance. Based on the structural equation modelling carried out, the result revealed that social networking impacts positively and significantly on the innovation performance of SMEs operating in the informal economy. Consequently, knowledge transfer that occurs during social networking that occurs between the SMEs and their international business associates help these SMEs to achieve technology breakthrough and new market breakthrough for their businesses. In line with this finding, existing research suggests that firms use social networking to develop connections through online communication, thereby empowering them to improve areas of concern, especially about technology adoption and enhancing their market competitiveness (Culnan et al., 2010; Gandomi and Haider, 2015).

## 7 Implications

This study has adequately shown that knowledge transfer significantly impacts on the innovation performance of small and medium enterprises operating in the informal economy of Nigeria. Specifically, this study has made an original contribution to knowledge management and international business literature by examining SMEs in the developing informal economies that are involved in international business relationships. Based on the findings from this study, it this study asserts that SMEs operators should intensify on R&D as a way of keeping themselves updated on the latest developments in the market and the changing customer trends, to innovate according to identified patterns. Also, SMEs operators should endeavour to establish social networks both among themselves, to facilitate learning and current innovations, and with their international associates. The benefits derivable from such global social networks would include innovating with price innovation mechanisms, for example acquiring products at lower lost. Another important implication from this study is that policymakers and investors should focus attention on encouraging and funding R&D and social networking activities to improve innovations among the SMEs in the informal economy.

Theoretically, this study has advanced reasoning in the knowledge management and international business literature about the impact of knowledge transfer and new product innovation performance of informal economy SMEs that are involved in international business relationships. The findings from this study establish a platform for further studies to be replicated in new developing economies and using a cross country analysis. The conclusions of this study apply to a larger group of SME operators in developing economies across the globe and such economies with similar characteristics with a growing informal economy. Hitherto, the literature consists of explanations relating to knowledge transfer among SMEs in international business from a developed economy perspective. The understanding of the characteristics and potentials of informal sector SMEs in international business relationships from a developing economy perspective has been an unfilled gap in the literature. Therefore, this study establishes empirical evidence about the relationship between knowledge transfer and new product innovation performance of informal sector SMEs in Nigeria.

## 8 Conclusion and further studies

In this study, the focus was on investigating the impact of knowledge transfer on innovation performance of small and medium enterprises in an informal economy. Following the findings from the structural equation modelling, the study concludes that knowledge transfer practices, especially R&D and social networking, have a significant impact on the innovation performance of informal economy SMEs that are involved in international business relationships. This study has advanced insights on the importance of stakeholders in the SMEs sector of informal economies to advance policies, funding and practices of R&D and social networking to improve SMEs innovative performance. Nonetheless, this study was limited to a single informal economy. Despite that Nigeria constitutes a substantial informal market in the global informal economy. Nevertheless, findings from future studies that will adopt a cross-country analysis may supply more robust insights.

## Declarations

### Author contribution statement

A. S. Ibidunni: Conceived and designed the experiments; Performed the experiments; Analyzed and interpreted the data; Contributed reagents, materials, analysis tools or data; Wrote the paper.

A. Kolawole: Conceived and designed the experiments; Contributed reagents, materials, analysis tools or data; Wrote the paper.

M.A. Olokundun and M. Ogbari: Contributed reagents, materials, analysis tools or data; Wrote the paper.

### Funding statement

This work was supported by 10.13039/501100012497Covenant University Centre for Research, Innovation and Discovery.

### Competing interest statement

The authors declare no conflict of interest.

### Additional information

No additional information is available for this paper.
